# What Mediates Fibrosis in the Tumor Microenvironment of Clear Renal Cell Carcinoma

**DOI:** 10.3389/fgene.2021.725252

**Published:** 2021-09-03

**Authors:** Wenbo Yang, Caipeng Qin, Jingli Han, Songchen Han, Wenjun Bai, Yiqing Du, Tao Xu

**Affiliations:** Department of Urology, Peking University People’s Hospital, Beijing, China

**Keywords:** clear cell renal cell carcinoma, fibrosis, PRLR, proliferation, biomarker

## Abstract

Previous studies have demonstrated that direct targeting of interstitial cancer-associated fibroblasts (CAF) and tumor fibrosis alone seemed to be an unpromising treatment option for malignant tumors. Therefore, it is necessary to further explore the mechanism of the influence of collagen and tumor fibrosis on the biological behavior of malignant tumors. The current study aimed to explore the effect of intratumor fibrosis on the prognosis of renal clear cell carcinoma (ccRCC) and its mechanism. With the bioinformatic analysis of The Cancer Genome Atlas (TCGA) database (*n* = 537), the study showed that high Collagen type I α 1 (COL1A1) mRNA expression indicated the poor prognosis of ccRCC patients compared with low expression ones. We further used the Two-photon-excited fluorescence (TPEF)/second harmonic generation (SHG) microscopy to determine the intratumor fibrosis of 68 patients with surgical resection of ccRCC and confirmed that a high fibrosis level in the tumor was associated with a poor prognosis compared with patients with low expression (Progression-Free Survival: *p* = 0.030). We further measured the protein chips of 640 cytokines in ccRCC specimens and found that several cytokines, including prolactin (PRL), were associated with the degree of fibrosis in the tumor, as confirmed by the prolactin receptor (PRLR) immunohistochemical method. In addition, the study showed that PRLR expression decreased significantly in the ccRCC compared with adjacent normal tissue (*p* < 0.05). Our research shows that low expression of PRLR predicted the poor survival of the patient. We used the Cell Counting Kit-8 experiment, the transwell and the plate clone formation assay to evaluate the role of PRL in the 7860 and the ACHN cell lines. We found that PRL promoted ccRCC cell proliferation and migration. JAK-STAT3 activation was found in the high prolactin expression group by mass spectrum analysis. This study delineated the fibrosis-based tumor microenvironment characteristics of ccRCC. PRL/PRLR may be involved in the fibrosis process and are essential prognostic risk factors for ccRCC.

## Introduction

The incidence of kidney cancer is increasing by 2% annually worldwide. Renal clear cell carcinoma (ccRCC) is the primary pathological type of kidney cancer, accounting for almost 80% of the cases (Cairns, 2010). The primary treatment for patients with localized ccRCC is tumor resection, and metastasis is found in approximately 20% of patients at their first visit. Even with early surgical treatment of ccRCC, approximately 30% of patients develop recurrence and metastasis. The 5-year survival rate of advanced ccRCC patients is only 23% (Rini et al., 2009). Therefore, there is a need to identify the molecular mechanisms underlying the proliferative and invasive phenotype of ccRCC to identify early diagnostic and prognostic markers and potential therapeutic targets.

Multiple studies have shown that cancer-associated fibroblasts (CAFs) play a more critical role in the occurrence and development of carcinoma (Nguyen et al., 2011; Ding et al., 2018; Wang et al., 2018). The stroma could also, in theory, promote vascular compression, and impede access to therapeutic agents. Fibrosis is associated with an extreme increase in the expression and deposition of collagen in the extracellular matrix (ECM), which can increase the biosynthesis of type I collagen by hundreds of folds (Zhang and Stefanovic, 2016; Ricard-Blum et al., 2018). Collagen is the most abundant extracellular matrix component and plays a significant role in ECM and fibrosis, regulating many histologically different tumor behaviors, including pancreatic and colon cancer and many stromal cells. Delahunt and Nacey (1987) divided fibrosis into mild, moderate and severe groups, and the results showed that fibrosis was not associated with the prognosis of ccRCC. Joung et al. (2018) applied more sensitive techniques the Two-photon-excited fluorescence (TPEF)/second harmonic generation (SHG) microscopy to quantify fibrosis in renal cancer, suggesting that intratumor fibrosis is associated with a higher histological grade of ccRCC. However, few studies have been performed on collagen when evaluating the biological behavior of ccRCC.

Furthermore, previous studies have demonstrated that direct targeting of interstitial CAFs and tumor fibrosis alone seemed to be an unpromising treatment option for malignant tumors (Narra et al., 2007; Özdemir et al., 2014). In addition, previous studies reported that some cytokines such as IL-1, IL-6, and IL-11, play an essential role in the progression of ccRCC (Knoefel et al., 1997; Chang et al., 1998; Pan et al., 2015a,b; Lee et al., 2019). However, few studies indicated cytokine assciation with the renal clear cell carcinoma matrix, especially the collagen abundance. Therefore, it is necessary to further explore the mechanism of the influence of collagen and tumor fibrosis on the biological behavior of malignant tumors. Encoding the pro-α1 chains of type I collagen, the primary type of collagen in tissue interstitial matrix, collagen type I α 1 (COL1A1) mRNA in tumors reflects the expression level of type I collagen (Boraschi-Diaz et al., 2017; Zhang et al., 2018; Kisling et al., 2019). With the bioinformatic analysis of The Cancer Genome Atlas (TCGA) database, the study showed that high Col1A1 mRNA expression indicated the poor prognosis of ccRCC patients compared with patients with low expression. We further used the TPEF/SHG technique to determine the intratumor fibrosis of ccRCC, and we assessed the degree of fibrosis in the tumor and the prognosis of the tumor based on the follow-up information. We further conducted a cytokine array analysis and explored the roles of differentially expressed genes in cancer cell behavior *in vitro*.

## Materials and Methods

### Clinical Specimens and TCGA Analysis

#### Human Tissues

Human ccRCC specimens were collected from patients who underwent surgical procedures. The studies were conducted following the ethical guidelines of the Declaration of Helsinki and were approved by the Peking University People’s Hospital Institutional Review Board. In addition, all surgically removed kidney tissues were collected following the Peking University People’s Hospital Institutional Review Board and were used for analysis under written informed consent from the patients.

The study database included 68 patients with pathologically proven ccRCC from Peking University People’s Hospital (PKUPH) who underwent surgery from 2011 to 2018. In addition, we extracted RNA-Seq data and clinical data from 537 ccRCC patients from TCGA and transcriptomic, proteomic, and phosphoproteomic characterizations of 110 ccRCC patients from the CPTAC data portal.

#### Quantification of Fibrosis Degree

As previous studies reported, the strength of the SHG signal is positively correlated with the collagen fibrils, and it can specifically show the abnormal deposition of collagen in the tissue. The TPEF microscopy can visualize tumor cells. By combining the two imaging technologies, the SHG/TPEF images are superimposed in a composite, which can directly show the morphology of collagen fibers and the surrounding tissue cells. The total deposition of all collagen fibers quantified by SHG/TPEF technology was deemed the tumor’s fibrosis level in the study, as described in previous studies (Wang Y. et al., 2019; Liu et al., 2020). According to the quantitative detection of tumor SHG/TPEF technology for total collagen level, ccRCC cases were divided into high fibrosis and low fibrosis groups by the median cutoff value.

#### Commercial Antibody Microarrays

Antibody microarrays were Human Cytokine Antibody Array Q640 (Catalog: QAH-CAA-640, QAA-CUST) quantitatively measure 640 human cytokines and Human Cytokine Antibody Array Custom (Catalog: QAA-CUST) purchased from RayBiotech. Compared with single-target ELISA and Western blot, the Antibody Array provides a broader view of protein activity. As previously described (Hou et al., 2014; Longhin et al., 2016), tumor specimens were homogenized, and cytokine standard dilutions were prepared after a series of processes. Furthermore, 100 μl of sample diluent was added to each well and incubated to block slides. After the buffer was decanted, 100 μl of either standard cytokines or the sample was added and incubated at room temperature for 1 h. Then, the samples were decanted, and the wells were washed five times. Next, 80 μl of the detection antibody cocktail was added to each well and incubated at room temperature for 1 h. Then, 80 μl of Cy3 equivalent dye-conjugated streptavidin was added to each well, and incubation was continued at room temperature for 1 h. The signals can be visualized using Axon GenePix, a laser scanner equipped with a Cy3 wavelength (green channel). Data extraction of the protein array was performed using GAL files, and microarray analysis was performed using microarray software (GenePix, ScanArray Express, ArrayVision, MicroVigene). Under the same experimental conditions, the experiment was repeated three times for the two groups of samples.

#### Tissue Processing and Immunostaining

Immunohistochemical staining and evaluation were conducted as described earlier. The effects of prolactin (PRL) on ccRCC cell proliferation, apoptosis, and metastasis were analyzed by corresponding cytological experiments, including Cell Counting Kit-8 (CCK8), colony-forming, Annexin V and 7-AAD staining, wound healing, and Transwell assays.

#### Analysis of Immune Infiltration in ccRCC Based on the TCGA and CPTAC Datasets

The CIBERSORT method is a computational method for inferring immune cell infiltration in a tumor based on tumor transcriptomes (Newman et al., 2015). ESTIMATE is also used to infer tumor purity and immune and stromal scores, quantifying the tumor’s stroma based on RNA-seq and proteome data (Yoshihara et al., 2013).

#### Prolactin Treatment

Recombinant human prolactin was purchased from R&D Systems Inc. Cells were serum-starved overnight and then cultured with 100 ng/mL human recombinant prolactin (hrPRL) for up to 12 h.

#### Cell Lines

The 786O and ACHN cell lines were obtained from ATCC in 2019. Cells used for the experimental study were passaged within 10–20 passages after reviving them from frozen vials. The cell lines were screened at early and late passages for Mycoplasma. The 786O cells were cultured in RPMI 1640 media (ATCC), and the ACHN cells were cultured in complete DMEM (ATCC) containing 10% FBS and 1% penicillin-streptomycin solution.

For the colony formation assay, 50–100 cells were seeded into six-well dishes and grown for 2 weeks. Then, the cells were fixed with 4% PFA for 30 min and stained with 0.1% crystal violet for 15 min.

#### Transwell Assay

A 24-well plate Transwell chamber product was used to perform the Transwell assay. Then, 5 × 10^4^ ccRCC cells in serum-free medium were seeded into the upper chamber (Corning, Inc. United States), while 10% FBS and 100 ng/mL hrPRL or the same volume of PBS was added to the lower chamber. After incubation for 24 h at 37°C, the cells in the upper chamber were removed. Cells in the lower chamber were fixed with 4% PFA for 30 min and stained with 0.1% crystal violet.

#### Immunohistochemistry (IHC) Staining for ccRCC

The cohort contained 68 ccRCC specimens and 30 adjacent normal tissues. The survival analyses were based on the detailed clinical data of these 68 cases. Briefly, paraffin sections were hydrated, embedded, and incubated with 3% H_2_O_2_ for 15 min. Tissues were then incubated with citrate buffer for antigen retrieval. Next, tissues were incubated with prolactin receptor (PRLR) antibody (Proteintech, catalog# 67292-1-lg) after blocking with 5% BSA followed by incubation with biotinylated secondary antibody. Next, tissues were incubated with HRP substrate solution for 30 min, followed by incubation with DAB substrate chromogen solution. Tissue slides were counterstained with hematoxylin, dehydrated, and mounted. The staining signal was calculated based on the scores of the staining intensity and the staining positive rate. The staining intensity was scored as follows: 0 points (negative), 1 point (weak), 2 points (moderate), and 3 points (strong). The positive staining rate was scored based on the positive cells as follows: 0 points (negative), 1 point (1–25%), 2 points (26–50%), and 3 points (51–100%). The formula calculates the PRLR total staining score: total score = staining intensity score × staining positive rate score.

#### Statistical Analysis

Statistical analysis was performed using SPSS, version 19.0 (IBM, Armonk, NY, United States), R software package, version 4.0.3 (R Foundation for Statistical Computing)^1^, the Timer website^2^ (Li et al., 2020) and GraphPad Prism 8. The correlation of tumor fibrosis with cytocines was verified in the CPTAC database by Spearman correlation analysis. To test the correlation of tumor fibrosis with cytokines we applied Student’s *t*-test and Kruskal-Wallis test and furtherly processed the data with Benjamini & Horchberg (BH) method to control for multiple hypotheses testing. In addition, Kaplan–Meier and rank-sum tests were used to construct the survival curve and to compare the differences between the curves. In all reports, a bilateral *p* < 0.05 was considered statistically significant.

## Results

### Demographic Characteristics of the Patients

ccRCC tissues from sixty-eight pathology-proven ccRCC patients were obtained from Peking University People’s Hospital (PKUPH). The characteristics of the patients are listed in Supplementary Table 1.

### Identification of Tumor Fibrosis as a Potential Prognostic Biomarker

**FIGURE 1:**
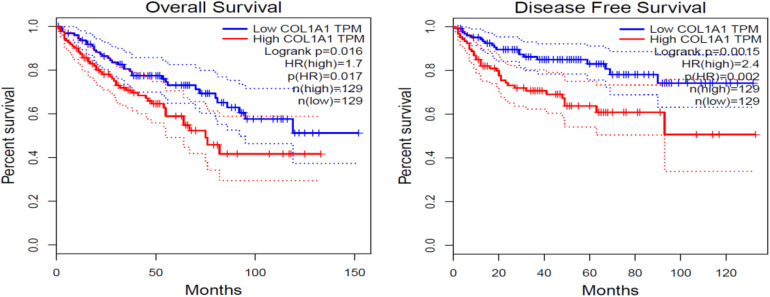
The impact of COL1A1 mRNA expression on the overall and disease-free survival of ccRCC patients. COL1A1 indicates the content level of type I collagen.

**FIGURE 2:**
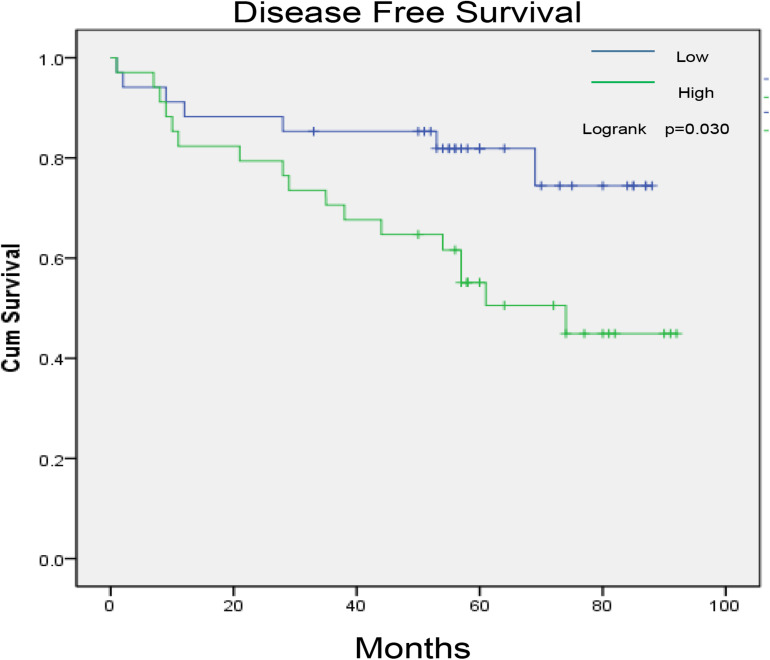
The impact of tumor fibrosis on the disease-free survival of ccRCC patients in the PKUPH cohort.

Col1A1 mRNA in tumors reflects the expression of type I collagen. Therefore, we grouped patients into high- and low-expression groups according to the quartile cutoff value. With the bioinformatic analysis of the TCGA-KIRC database (*n* = 537), the survival curves in Figure 1 showed that high Col1A1 mRNA expression indicated poorer overall survival and disease- free survival (PFS) of ccRCC patients than low expression ones (*p* < 0.05). As Figure 2 showed, we further used the TPEF/SHG technique to determine the intratumor fibrosis of 68 patients with surgical resection of ccRCC and confirmed that a high fibrosis level in the tumor was associated with a poor prognosis compared with low expression (PFS: *p* = 0.030).

### Identification of Prolactin as the Essential Cytokine Involved in Tumor Fibrosis in ccRCC

As Supplementary Table 2 showed, we conducted an unbiased cytokine array analysis and found nine upregulated cytokines in ccRCC tissues with high fibrosis compared with those in tissues with low fibrosis (*n* = 11), including brevican, cerebral dopamine neurotrophic factor (CDNF), prolactin, GRO, IL-17, IL-6Rα, IL-7, IL-9, and presenilin 1 (*p* < 0.05). Other cytokines including IL-1 and IL-11 had no significant association with collagen abundance in the study (*p* > 0.05). As the *p*-value was adjusted, only seven cytokines remained statistical significance, including brevican, presenilin 1, prolactin, CDNF, IL-17, IL-9, and IL-6Rα (*q* < 0.05). Among these, PRL/PRLR was observed to have a specific association with tumor fibrosis, and PRLR was confirmed by IHC (*n* = 68, rho = −0.281, *p* = 0.020).

### PRL Is Upregulated in ccRCC Samples

**FIGURE 3:**
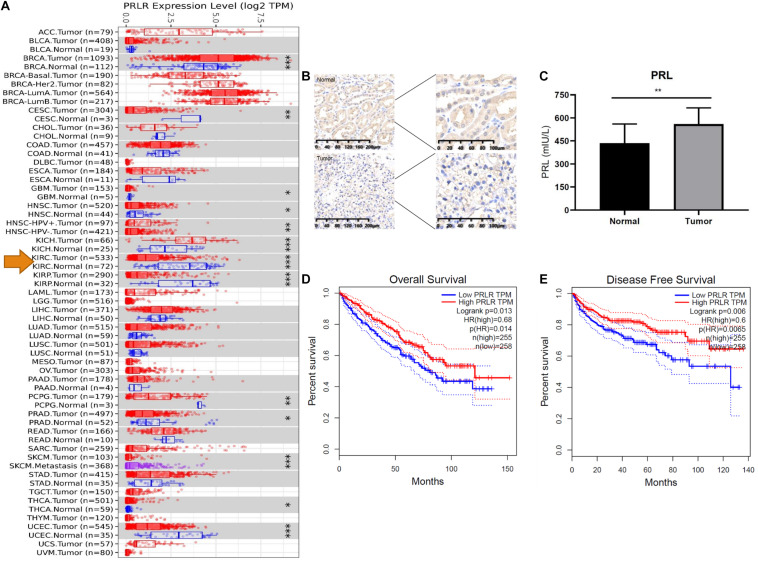
PRL/PRLR signal in ccRCC. **(A)** PRLR expression is significantly decreased in tumors compared with paracancerous tissues. Tumors and paracancerous tissues were shown in red and blue dots, respectively. The tumors and paracancerous tissues of a certain cancer was in the same gray box (*p < 0.05; **p < 0.01; ***p < 0.001). **(B)** PRLR was stained with IHC in the tumor and normal tissue. **(C)** PRL was quantified with ELISA. **(D)** The impact of PRLR on the overall survival of ccRCC patients in the TCGA cohort. **(E)** The impact of PRLR on the disease-free survival of ccRCC patients in the TCGA cohort.

We compared the mRNA expression of PRLR in cancer tissues and paracancerous tissues of various malignant tumors. As shown in Figure 3A, the expression of PRLR in most cancer tissues was significantly lower than that in paracancerous tissues, including ccRCC, cervical squamous cell carcinoma, endocervical adenocarcinoma, and uterine corpus endometrial carcinoma. To determine whether PRL signaling occurs in ccRCC, we first analyzed the expression of PRL and PRLR in human ccRCC tissues and cell lines. IHC using ccRCC patient samples indicated a significant decrease in PRLR, and enzyme-linked immunosorbent assay suggested an increase in PRL in cancerous tissue compared with paired adjacent normal tissue (Figures 3B,C). Quantification of concentrated culture media from the cell lines by enzyme-linked immunosorbent assay indicates that all cells secrete prolactin. However, the amount varies in a cell line-specific manner after 24 h. hrPRL in ccRCC was upregulated compared with normal paracancerous tissues, suggesting a significant role in the pathogenesis of ccRCC.

### Tumor-Derived Prolactin Promotes the Tumor Progression of ccRCC Cancer Cells

To further explore the potential influence of PRL/PRLR on OS and DFS, we performed Kaplan-Meier survival analysis with data from the TCGA database. As Figures 3D,E showed, PRLR was associated with a favorable prognosis, including OS and DFS of ccRCC (*p* < 0.05). Interestingly, our study indicated that PRLR expression was significantly associated with overall survival rates and disease-free survival rates in the TCGA (*n* = 534) and PKUPH cohorts (*n* = 68).

### Prolactin Promotes the Proliferation and Migration of Renal Carcinoma Cells

**FIGURE 4:**
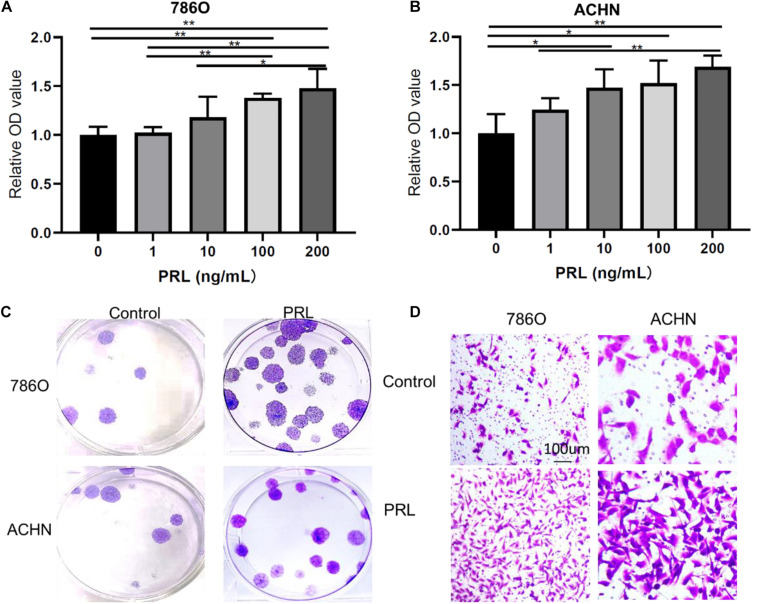
Overexpression of PRL in ccRCC. **(A)** Prolactin can stimulate the proliferation of the 786O cell line. **(B)** Prolactin can stimulate the proliferation of the ACHN cell line. **(C)** Prolactin treatment promotes the clonality of the 786O and ACHN cell lines. **(D)** Prolactin treatment promotes the migration of the 786O and ACHN cell lines (*p < 0.05; **p < 0.01).

*In vitro*, PRL overexpression can significantly induce cell proliferation and migration and inhibit cell apoptosis. Therefore, as the first step to study the PRL/PRLR axis *in vitro*, we examined two ccRCC cell lines, 786O and ACHN, which expressed PRLR. While investigating the effects of PRL/PRLR on the proliferation of 786O and ACHN cells treated with PBS control and 100 ng/mL prolactin for 24 h, CCK-8 assay and colony formation assay results suggested that PRL drastically promoted ccRCC cell proliferation (Figures 4A,B). In addition, Transwell migration assays showed that overexpression of PRL promoted the migration of ccRCC cells (Figures 4C,D).

### Prolactin Promotes STAT3 Activation in ccRCC

To determine the mechanism by which prolactin promotes ccRCC, we obtained phosphorylation and transcriptome databases of 110 renal cancer patients with CPTAC and found that PRL was positively correlated with *p*-STAT3 (*p* = 0.03, rho = 0.326) and *p*-JAK3 (*p* = 0.014, rho = 0.812).

### Correlation Between CAF and PRL in ccRCC

To explore the relationship between cytokines and the proportion of CAFs in tumors, we used the EPIC method based on the TCGA dataset. The study found that the CAF proportion was significantly negatively correlated with PRLR in various malignant tumors, including ccRCC (rho =−0.11,*p* < 0.001). In contrast, presenilin 1 or IL-7 remained not statistically significant (*p* > 0.05).

## Discussion

The importance of the tumor microenvironment in tumor initiation and progression has been widely recognized. Although most cytokines and their receptors are expressed in microamounts in tumors, they are essential in regulating the tumor microenvironment (Wang J. et al., 2019). First, this study delineated that high tumor fibrosis might predict poor prognosis of patients through SHG/TPEF quantitative assessment of renal cancer tissue fibrosis grade. Second, this study combined protein microarray analysis to look for differentially expressed genes and found a significant correlation between PRL/PRLR and fibrosis in tumor tissues. Recently, Col1A1 mRNA expression was reported to be associated with bone metastases in ccRCC and breast cancer (Liu et al., 2018; Gao et al., 2020). The study confirmed the essential role of Col1A1 and tumor fibrosis in the prognosis of ccRCC. Qin et al. (2020) elaborated that ccRCC patients with higher levels of tumor fibrosis detected by SHG/TPEF were associated with an unfavorable prognosis, but there was no further study on its molecular mechanism. The study further combined SHG/TPEF with cytokine array analysis to explore the molecular mechanisms of tumor fibrosis.

Although the study confirmed that intratumoral fibrosis predicted a poor prognosis for ccRCC, previous studies have demonstrated that direct targeting of interstitial CAFs and tumor fibrosis alone does not seem to be a good treatment option for malignant tumors (Narra et al., 2007; Özdemir et al., 2014; Rhim et al., 2014). Therefore, it is necessary to understand the underlying causes of tumor fibrosis and their roles in the tumor microenvironment. Therefore, bioinformatics analysis and immunohistochemistry methods were used to confirm that the PRL/PRLR signaling pathway can promote the progression of ccRCC *in vivo* and *in vitro*. Thus, this study is the first to indicate that PRL might regulate tumor fibrosis and the progression of ccRCC. Similarly, Tandon et al. (2019) concluded that PRL played an essential role in tumor fibrosis and that it promoted pancreatic cancer progression *in vivo* and *in vitro*.

In this study, we found that the expression of PRL in ccRCC tissues was much higher than that in paracancerous tissues and that it was related to a poorer prognosis for patients with ccRCC. PRL was first noted in ccRCC as a result of a case report in which physicians accidentally found a kidney cancer patient with hyperprolactinemia, and their prolactin levels returned to normal after tumor resection (Stanisic and Donovan, 1986). Previous studies suggested that PRLR knockout T lymphocytes had an increase in autocrine PRL and that PRL had a negative feedback regulation on PRLR expression. The study also indicated a negative feedback relationship between PRLR and PRL in renal cancer. Recently, PRL was demonstrated to be positively associated with PSEN1 and presenilin enhancer (Neradugomma et al., 2014). Our study indicated that PRL and PSEN1 were associated with tumor fibrosis and that prolactin might be the key cytokine regulating tumor fibrosis.

For patients with ccRCC with poor immunotherapy effects, it is the most appropriate choice to change our thinking and develop drugs targeted at the fibrosis-related microenvironment of tumors. Our results provide some potential targets of stromal therapy that are worth further research and may potentially promote ccRCC treatment in the future. However, due to the high price of antibody microarrays and the relatively small number of samples in the screening, we might have ignored some other cytokines involved in tumor fibrosis. In addition, the experimental results of this study need to be further confirmed by knockdown and overexpression of PRL/PRLR.

## Conclusion

PRL regulates tumor fibrosis, plays a promoting role in the growth and metastasis of ccRCC and may be a potential therapeutic target for ccRCC intervention. In addition, therapeutic targets to the stroma, another essential component of the tumor microenvironment, may be an instrumental complement to immunotherapy for ccRCC.

## Data Availability Statement

The original contributions presented in the study are included in the article/Supplementary Material, further inquiries can be directed to the corresponding author/s.

## Ethics Statement

The studies involving human participants were reviewed and approved by Peking University People’s Hospital Institutional Review Board. The patients/participants provided their written informed consent to participate in this study. Written informed consent for publication was obtained from all participants.

## Author Contributions

WY, CQ, and TX conceived and designed the study. JH, YD, and SH performed data curation. CQ and YD performed formal analysis. WY and WB conducted methodology. TX performed supervision, reviewed, and edited the manuscript. WY and CQ wrote the manuscript. All authors read and approved the manuscript.

## Conflict of Interest

The authors declare that the research was conducted in the absence of any commercial or financial relationships that could be construed as a potential conflict of interest.

## Publisher’s Note

All claims expressed in this article are solely those of the authors and do not necessarily represent those of their affiliated organizations, or those of the publisher, the editors and the reviewers. Any product that may be evaluated in this article, or claim that may be made by its manufacturer, is not guaranteed or endorsed by the publisher.

## Footnotes

1
http://www.r-project.org/

2
http://timer.comp-genomics.org/
