# Supplementary material for: What Mediates Fibrosis in the Tumor Microenvironment of Clear Renal Cell Carcinoma
Source: Front Genet. 2021 Sep 3;12:725252. doi: 10.3389/fgene.2021.725252 (PMC8446447; doi:10.3389/fgene.2021.725252)
Supplement: Supplementary file 1 [file Table_1.docx]

**Supplementary Table 1**. The clinical characteristics of PKUPH enrolled cases

|  | PKUPH (n=68) |
| --- | --- |
| Age at surgery (years) | 55.9 ± 13.4 |
| Gender |  |
| Female | 16 |
| Male | 52 |
| p-Stage |  |
| 1-2 | 44 |
| 3-4 | 24 |
| Fuhrman grade |  |
| 1-2 | 50 |
| 3-4 | 18 |
