# Supplementary material for: What Mediates Fibrosis in the Tumor Microenvironment of Clear Renal Cell Carcinoma
Source: Front Genet. 2021 Sep 3;12:725252. doi: 10.3389/fgene.2021.725252 (PMC8446447; doi:10.3389/fgene.2021.725252)
Supplement: Supplementary file 2 [file Table_2.docx]

**Supplementary Table 2.** Cytokines involved in tumor fibrosis of ccRCC

| FRC | Genes | IF(n=11) | HF(n=11) | p | q |
| --- | --- | --- | --- | --- | --- |
| Brevican | BCAN | 4.29±7.80 | 16.15±14.50 | 0.034 | 0.034 |
| CDNF | CDNF | 25.62±23.48 | 53.94±45.80 | 0.031 | 0.046 |
| GROa | CXCL1 | 387.35±694.99 | 1073.69±620.80 | 0.001 | 0.089 |
| IL-17 | IL17A | 6.21±7.41 | 13.39±6.31 | 0.021 | 0.048 |
| IL-6R | IL6R | 92.63±46.94 | 135.68±47.43 | 0.027 | 0.050^#^ |
| IL-7 | IL7 | 7.59±7.13 | 16.29±9.16 | 0.015 | 0.068 |
| IL-9 | IL9 | 569.87±562.09 | 1666.35±1146.83 | 0.016 | 0.049 |
| Presenilin 1 | PSEN1 | 1.00±1.06 | 2.09±1.16 | 0.034 | 0.039 |
| Prolactin | PRL | 17.58±21.42 | 42.74± 42.68 | 0.033 | 0.042 |

BCAN: brevican, CDNF: cerebral dopamine neurotrophic factor, GROa: chemokine (C-X-C motif) ligand 1 (melanoma growth stimulating activity, alpha). #: The q-value of IL-6R was 0.0498 when keeping four digits after the decimal.
